# The Political Theory of Modus Vivendi

**DOI:** 10.1007/s11406-016-9800-1

**Published:** 2017-01-12

**Authors:** Peter Jones

**Affiliations:** School of Geography, Politics and Sociology, Newcastle University, Claremont Tower, Newcastle upon Tyne, NE1 7RU UK

**Keywords:** Modus vivendi, Liberalism, Liberal moralism, John Horton, John Rawls, Ideal theory, Non-ideal theory, Legitimacy, Scepticism

## Abstract

One of John Horton’s most original and significant contributions to political theory is his development and exploration of the political theory of modus vivendi (MV). I examine what Horton understands a MV to be, what sort of theory he intends the political theory of MV to be, and why he believes a MV to be the best we can reasonably hope for. I consider how far his notion of MV matches the reality of contemporary political systems and whether ‘liberal moralism’ is quite as divorced from reality or as devoid of practical consequence as his political theory of MV would have us believe.

John Horton is much admired by his peers for his fair-mindedness, his sagacity, and his strong sense of professional obligation. Those qualities have made him everyone’s favourite referee, assessor and external examiner. His worldly wisdom, breadth of learning, clear-headedness, and sobriety of judgment are also evident in his work as a political theorist, but, as a political theorist, he has used those qualities to more controversial effect. Horton is a prominent and trenchant critic of the dominant form that political theory has assumed during the last four decades, a period roughly co-extensive with his own career as a political theorist. He has used his elegant, measured and carefully modulated prose to deliver a swingeing assault on contemporary liberal political theory. He is not antipathetic towards a liberal political order. On the contrary, he is favourably disposed towards that order and also towards the liberal tradition (2009, 30; 2010a 447; 2011a, 131). His criticism is principally directed at the form that liberal political theory has taken since the publication of John Rawls’s *A Theory of Justice* (1971). That idealised and moralised form of political theory, which Horton brands ‘liberal moralism’, runs counter to the scepticism that he himself brings to the study of politics and to his belief that political theory has to stay close to political reality if it is to have any value.

For those of us who are sympathetic to the endeavours of Rawls and of other liberal political theorists to deal with the issues raised by pluralism, a particularly irritating feature of many of the critics is the entirely negative character of their own efforts. They find fault with liberal thinking but then either walk away from the issues that contemporary liberals are trying to resolve or pretend that those issues do not exist. Horton is an honourable exception to that tendency. Indeed, part of his complaint against ‘liberal moralism’ is its failure fully to recognise how problematic and unamenable is the contemporary pluralism for which it ostensibly provides. In his own political theory of modus vivendi he has sought to replace liberal moralism with an understanding of political life that takes division and dissensus more seriously and to provide a more realistic account of the sort of rapprochement that division and dissensus permit.

In this article, I investigate what Horton understands by a modus vivendi (hereinafter ‘MV’), what kind of political theory he intends the political theory of MV to be, and what leads him to suppose that MV is the best for which we can realistically hope. I then comment on how far his notion of MV matches the reality of the contemporary political world, particularly the reality of political systems whose foundations are not embroiled in dispute, and consider whether contemporary political philosophy is quite as divorced from reality and as devoid of practical consequence as his political theory of MV would have us believe.

## Modus Vivendi

How, then, does Horton understand a MV? A MV assumes a situation of conflict, potential if not actual (2007, 55; 2011a, 121–4; 2011b, 292–4). The term ‘modus vivendi’ translates literally as ‘way of living’ but in contemporary political usage it describes a way of living together that a population has achieved in spite of features of itself that might bring its members into conflict with one another. Very often that conflict will arise from differences, such as differences of interest, belief, value, ideology, culture, identity, faith or ethnicity. MV is most commonly conceived as an arrangement that recognises and provides for differences of those sorts. But, as Hobbes observed, even a population that possesses identical preferences can find itself embroiled in conflict if their identical preferences place them in competition for scarce resources. Thus, even a homogeneous population may need a MV. Even so, MV is nowadays more commonly associated with difference and is invoked most frequently as a device for managing the differences that characterise modern societies, especially when those differences generate ‘deeply divided’ societies.

Horton also associates MV with contingency, compromise, particularity and circumstance (2007, 51–2; 2010a, 441–2; 2011a, 123–7). An MV describes an arrangement that a number of parties have negotiated or evolved that enables them to live together peacefully, given their particular characteristics and differences and given the realities of their particular situation. It is not the realisation of a general ideal. On the contrary, it will typically be an arrangement that has been cobbled together in response to the realities in which the parties find themselves, and one that takes advantage of whatever they can find in themselves, in one another and in their given circumstances that facilitates peaceful coexistence. For all those who are parties to it, a MV will be a compromise. MV therefore has a ‘second best’ quality, even though practically it may be the best that is feasible. We can therefore think of MV as something that, in conception, is sandwiched between other sorts of arrangement that are either less than or more than itself, and we can get a fix on the idea of MV by considering what it is not.

What order of things, then, falls short of MV? Horton is clear that an arrangement in which a regime simply tyrannises over and supresses a population, or uses its power to enable one part of a society to impose its will unilaterally upon another, cannot count as a MV (2010a, 439, 442; 2011a, 124–5, 128). Tyranny or mere domination is at odds with the very idea of the different parts of a population ‘living together’ which the term MV is designed to capture, even though the life they make together may be no more than a political life. So while Horton associates MV with political realism, he does not associate it with a realism in which just anything goes and he is as dismissive of ‘false realists’, who pretend that politics is only ever about power and interest and who valorise brutality and amoralism, as he is of liberal moralists (2010a, 441–2).

For Horton, consent to, or acceptance of, a MV by the parties to it is crucial to its being a MV (2007, 52; 2010a, 438–9, 443; 2011a 124–5, 128). A MV embodies compromise and an arrangement is a compromise only if each of the parties to it concedes something and only if each agrees to it (Jones and O’Flynn 2013). That feature of MV raises the thorny question of what is to count as consent or acceptance or agreement. It is part of the political theory of MV that we should not set the threshold for meeting these requirements too high. The political theory of MV stresses that the circumstances for MV are those of disagreement, conflict, untidiness and complexity, in which all of the parties have to settle for something less than they would ideally like. They may also have to settle upon a MV under conditions of duress, disadvantage and inequality. So the consent or acceptance that Horton requires for MV is very much less than consent given under conditions of freedom and equality of the sort demanded by some liberals, at least for theoretical purposes.

While consent or acceptance is therefore a crucial feature of MV, it is consent or acceptance whose definition remains imprecise (Horton 2010a, 443). In real-world contexts of the kind Horton means to address, it is hard to be precise about these things, but lack of precision does not mean there is no contrast to be made. For example, there would be no justification for describing as a MV the historical treatment of indigenous peoples by settler populations, in which settlers fought against, suppressed and often eliminated indigenous populations in large numbers. By contrast, the arrangements that later came to be established between settler populations and indigenous peoples, and that now obtain in countries like Canada, Australia and New Zealand, might be reasonably described as a MV, at least in some instances.

If tyranny and repression sink beneath a MV, what rises above it? How good can a MV be before it becomes more than a MV? Horton’s political theory of MV is conceived in opposition to Rawls’s political conception of justice and especially in opposition to that conception conjoined to Rawls’s claim that it can become the focus of an overlapping consensus amongst the different and conflicting comprehensive doctrines that the members of his model society embrace. So, at its upper end, the notion of MV is to be distinguished from a society all of whose members regard its arrangements as just and who experience no conflict between their comprehensive doctrines and their society’s public arrangements. In Rawls’s just society, ideally conceived, there is no element of compromise or regret for any of its members, at least in relation to the society’s basic structure and constitutional essentials. All are thoroughly wedded to the society as a *just* society and none would wish it to be different.

That Rawlsian ideal is more than a MV and is the sort of ideal that Horton intends his model of MV to challenge. He rejects Rawls’s ideal not because he finds it undesirable or morally wrong but because he believes it unrealistic, impossible, utopian, and so politically irrelevant (2010a, 433–7; 2011a, 125–7). It demands more than we can ever reasonably expect given the realities of political life.

Horton does argue, however, that a MV can be more and better than the account of it given by Rawls himself. Rawls (1993, 147–8) understands a MV to rest upon a mere balance of political forces and to be grounded in nothing more than the self-interest of those who are party to it. Because it is rooted in self-interest only, each party will be ready to defect from it as soon as the balance of forces changes such that adherence to the MV ceases to be to its advantage all things considered. For that reason, Rawls deems a MV inherently unstable.

Horton, by contrast, insists that a MV need not be constructed only on the basis of naked self-interest. It can be constructed from whatever the parties bring to the situation in which they find themselves.

… a modus vivendi can be arrived at by drawing on whatever resources – moral, intellectual, cultural, pragmatic , etc., as well as self-interest – are available in helping the parties to reach it. A modus vivendi is not therefore to be understood, as Rawls does, simply as a balance of political forces. … A modus vivendi is a practical accommodation that can be built around any number of factors and be accepted for a variety of reasons [moral as well as prudential] by those who are parties to it. (2010a, 439–440; see also 2011a, 124, 126–7; 2011b, 295–8.)

However, we need to be careful about just what the claim is here. It is not that, insofar as people bring moral beliefs or bits of moral belief to the construction of a MV, they will somehow moralise it from a third party perspective, particularly the perspective of the political theorist of MV. Rather, Horton’s claim here is entirely instrumental. It is that the moral beliefs of the parties to a MV, particularly the beliefs they share, may facilitate its construction and enhance its stability. Insofar as beliefs have this benign effect, their influence will be welcomed by the political theorist of MV and they should be welcomed by those who are seeking the MV. But the ‘helpfulness’ of moral beliefs (or beliefs of other sorts, such as religious or political beliefs) need have nothing to do with their intrinsic merit as beliefs; nor does their helpfulness hold out the prospect that a MV might be based on either a single intersubjective morality, or a jointly recognised objective morality, that removes all need for accommodation and compromise. And, of course, both moral beliefs and beliefs of other sorts may prove more of a hindrance than a help in the construction of a MV, since brokering conflicting beliefs is often more difficult than brokering conflicting preferences or interests (cf. Horton 2011a, 127, 129–32). So Horton’s suggestion that the parties can draw on more than mere self-interest in constructing a MV does not imply that the MV they construct will have a more satisfying moral character than the purely self-interested version that Rawls describes.

It is something else that would seem to give a moral quality to Horton’s version of MV, which takes us back to the idea of acceptance and, more particularly, to the idea of legitimacy. Horton says that we can expect, or at least reasonably hope, that the parties to a MV will come to regard it as *legitimate* (2010a, 438–40, 443; 2011a, 128). Indeed, a MV will ordinarily become entrenched in a society’s laws, institutions and constitution all of which can be deemed legitimate by the society’s members (2010a, 440; 2011a, 125). It is that idea of legitimacy that gives Horton’s MV a normative dimension that is absent from Rawls’s version of MV. It is also the parties’ commitment to the legitimacy of their MV that, along with their experiencing the benefits of MV, provides the main ground upon which Horton rejects Rawls’s claim that a MV must be unstable (2007, 49–51; 2010a 439–41).

The introduction of that idea of legitimacy raises the issue of how we are to distinguish a MV, particularly a good MV, from the kind of ideal Rawls envisages. If every section of a society endorses the legitimacy of its political arrangements, how does the society remain fundamentally different in character from Rawls’s just society? Horton’s requirement of acceptance and his emphasis upon legitimacy do seem to add a deontological dimension to his notion of MV. If we ask why people would enter into a MV, given the deep and serious nature of the conflicts between them, Horton’s main answer is to achieve the goods of peace and security and all the multifarious goods for which peace and security are preconditions (2007, 54–5; 2010a 438, 444; 2011a, 125). Legitimacy may therefore have only instrumental value for the political theory of MV: it may matter only if and because it assists in bringing about and maintaining peace and security.

However, valuing legitimacy only in that way is hazardous, since tyranny and repression can perform as well as, if not better than, a MV as instruments of peace and security. That is no mere logical possibility: consider the conflicts that developed in the former Soviet Union and the former Yugoslavia once the yoke of communist rule was cast off, or what happened in Iraq following the toppling of Saddam and the Ba’athist regime, or what has happened and what might yet happen following the displacement of authoritarian regimes in Libya and Syria. Peace and security are often achieved more readily, and more effectively and reliably, through a repressive regime than through a cobbled-together and potentially fragile MV.

Horton may therefore insist on legitimacy as a feature of MV not only for its instrumental value but also because it is a minimally *right* requirement. He may hold that it is wrong and unacceptable that people should have to live under a regime that is devoid of legitimacy (Horton 2012, 131). But how then does his conception of MV, particularly MV at its best, differ categorically from Rawls’s ideal? The answer seems to lie in two related considerations.

One is the question of what it is that renders an arrangement legitimate. Should the legitimacy of a regime depend upon its intrinsic quality or upon the way in which it is regarded by its population? The thinking of Rawls and many other liberal philosophers approximates more closely to the first of these options. Although they often invoke consent as a condition of legitimacy, their test of legitimacy relates primarily to a regime’s moral quality. Consent figures as an adjunct to that test – given the quality of the regime, should it command the consent of a population or would it command their consent if the population were ‘reasonable’? In other words, the relevant consent is a consent that people ought to give rather than a consent they actually give. For Horton (2012), that gets things the wrong way round. The legitimacy of a regime turns on how it is regarded by its population, although that is not to say that a regime’s legitimacy is conferred upon it by the consent of its population as ‘consent’ is understood in voluntarist theories of political legitimacy. Rather, the legitimacy of a regime depends upon whether it meets the criteria that its population reckon provide the relevant tests and these tests need not include the population’s own consent, although presumably they could. So the relevant matter is not so much a population’s consent as, in David Hume’s phrase, their ‘opinion of right’ (Hume 1963). Standards of legitimacy will be embedded in the culture, circumstances and traditions of a population and so will vary with time and place. Thus, Horton does not hold that legitimacy is conferred upon a regime only by the consent of those who are subject to it and certainly not by the individually given consent demanded by Lockean contract theory. Nor does he propose a single criterion or set of criteria for legitimacy. Even so, for the political theory of MV itself what makes a MV legitimate is its being deemed acceptable by those who are party to it. In that sense, if only in that sense, the legitimacy of a MV might be said to rest upon the ‘consent’ or ‘acceptance’ of those who are subject to it.

A second consideration is that, for Horton, legitimacy is a less demanding and less precise notion than justice, and a population can accept an arrangement as legitimate even though they reckon it less than fully just (2010a, 439; 2011a, 128; 2012, 134–7). That decoupling of justice from legitimacy is essential if there is to remain a clear distinction between MV and Rawls’s political conception of justice. In a MV the parties are always less than completely satisfied with their condition. From their point of view, the MV is always less than fully just or fair and always less than what they really want it to be or believe it should be, even though they may deem it legitimate. A MV therefore never fully resolves conflict in the way that Rawls’s political conception of justice aspires to. While Rawls’s arrangement does not remove conflict amongst people’s different comprehensive doctrines and their associated conceptions of the good, it does deal with that conflict in a way that is just and that all citizens, if they are reasonable, will recognise as just. Thus, unlike the parties to a MV, Rawls’s citizens have no reason to be less than fully satisfied with their society. However, for Horton, while we may posit a political condition that is superior to MV, in reality no polity will supersede a MV: ‘even the best polities will never be entirely just (whatever one’s understanding of justice)’ and most will have other serious ethical failings (2010c, 163; also 2010a, 438). Indeed, he doubts ‘that we can even *imagine* a wholly just liberal state’ (2010a, 436, his emphasis; see also 2005, 31–3; 2007, 51–2).

## The Political Theory of Modus Vivendi

That is not to say that Horton regards justice as a matter of no consequence. He does worry about it and particularly the charge that a MV may be significantly unjust (2007, 52–4). The reassurance he offers is that a MV requires the parties’ acceptance and they are unlikely to accept their own radically unjust treatment (2010a, 439; 2011a, 128). But that thought is merely a consolation. A MV does not have to be just and tolerating a degree of injustice is likely to be a price the parties have to pay to secure a MV. Indeed, different and clashing conceptions of justice are likely to be part of the very conflict for which a MV has to provide (2003, 21; 2007, 53–4).

My aim so far has been simply to give a brief overview of the idea of MV as it is sketched in various of Horton’s writings. That is the order of things presented by his political theory of MV. But what sort of ‘political theory’ is it? Does Horton intend his portrait of MV to provide us only with an interpretation of the contemporary political world that exposes Rawls’s political conception of justice as hopelessly adrift from reality? Or is its purpose to present and defend a possibility – the possibility that a coercive and oppressive regime is not the only alternative for a population if it cannot come up to the standard set by Rawls’s just society? Does it, in other words, present us with an ideal, albeit an ideal of an avowedly un-utopian sort? MV may be more realistic and therefore less ambitious in aspiration than idealised models of the just society, but it can still present us with a goal that is achievable and one whose achievement we should welcome and value. So, rather than merely holding up a mirror to reality, Horton’s political theory of MV may be no less normative in purpose than the prescriptions of liberal moralism.

In fact, the normative status of Horton’s MV is less than entirely clear and uncertainty about that status arises at several crucial points in his account. One such point is the distinction he makes between MV and tyrannous regimes that maintain their position merely through fear and coercion. Inevitably there is a degree of fuzziness in the boundary that separates MV from merely repressive regimes, particularly since MV comes in different degrees and will, like any real political regime, make use of coercion itself (2011a, 124–5). That fuzziness is not the issue here. There will still be plenty of cases in which it is clear that a regime falls either above or below the line. But how are we to understand what is at issue when we make that distinction? Earlier I described a MV as sandwiched between arrangements that are either ‘more’ or ‘less’ than itself. That characterisation suggests a normative hierarchy in which MV ranks more highly than tyrannical or repressive regimes. But it may be no part of Horton’s purpose to suggest such a hierarchy. He may exclude tyrannies from MV simply because they fall outside its conceptual limits. If a defining feature of MV is broad acceptance by those whom the MV governs, a tyranny will not be a MV simply because ‘by definition, a tyrannical political settlement is not one that is broadly accepted by those subject to it’ (2011a, 128). While it would be odd not to regard a coercive regime as morally inferior to one that is embraced by those whom it governs, it may be no part of the political theory of MV to make that sort of judgement. Indeed, the realism that undergirds Horton’s political theory might allow that there can be circumstances in which a repressive regime may be the only realistic possibility or, as we noted earlier, circumstances in which a repressive regime will deliver the goods of peace and security more adequately than any MV that is realistically possible in those circumstances.

A similar ambivalence of normative status is apparent when we turn to the related issue of legitimacy. Horton, as we have seen, treats legitimacy as a central feature of MV. He insists, of course, that the degree of legitimacy a MV can secure will be contingent on the circumstances in which it has to be forged and also that we should be realistic in the standard that we set for legitimacy. But legitimacy would still seem, of necessity, to be normatively superior to illegitimacy and greater legitimacy to be preferable to less.

In fact, the concept of legitimacy is itself ambiguous. When we observe that a regime enjoys legitimacy, we may do so in any entirely empirical or sociological spirit: we may mean only that the regime is, as a matter of fact, accepted as rightful by its population. If we use the concept in that way, we need imply nothing about whether the regime is indeed rightful and ought to be complied with by its population. We merely note a socio-political fact. Let’s call this ‘empirical legitimacy’. Alternatively, when we describe a regime as ‘legitimate’, we may do so in a wholly normative spirit: we may mean to affirm that the regime is indeed rightful and therefore deserving of its population’s compliance. Let’s call that ‘normative legitimacy’. When Horton describes a MV as legitimate, or indicates that different MV may enjoy different degrees of legitimacy, which of these two senses of legitimacy does he intend? The Kantian and libertarian accounts of political legitimacy that he opposes are clearly normative in character and are normative in ways that are quite independent of empirical legitimacy. Horton, by contrast, aims ‘to restore the connection between political legitimacy and the beliefs and attitudes of those subject to it’ (2012, 141). For him, the test of a regime’s legitimacy is whether it is, as a matter of fact, attributed with the right to rule by those whose regime it is. That test seems to substitute an empirical conception for the normative conceptions proposed by Horton’s opponents. If it does, it is in harmony with his desire to substitute realism for moralism. It offers us not merely a theory of legitimacy that differs substantively from those offered by Kantians and libertarians but also a different *kind* of theory – a ‘theory’ that deals with the issue of legitimacy in a quite different way. It shifts the focus from norms fashioned by political theorists to the empirical matter of how a regime is conceived by its own population. Once again, therefore, we have reason to query the normative import of the political theory of MV.

Yet Horton goes on to deny that his account of legitimacy is purely descriptive and insists that the idea of political legitimacy, in distinguishing between legitimate and illegitimate power, is ‘inherently normative’ (2012, 145). It may be therefore that his account of legitimacy is not only empirical. He may intend the empirical test of legitimacy also to be the normative test: if, as a matter of fact and for whatever reason, a population deems its regime to be legitimate, that is reason enough to deem the regime normatively legitimate. But it cannot be that simple, since Horton also remarks that there is space in his account ‘for people to be mistaken or deluded about the legitimacy of their political institutions’ (2012, 145).

This ambivalence on how we are to think of legitimacy in relation to MV bears on its significance for MV. If the legitimacy relevant to MV is normative, it introduces a deontological element into MV: the legitimacy that is a necessary feature of MV implies that MV enjoys a rightness that sub-MV arrangements do not. Our thinking on legitimacy may have to be tempered by the realities of politics, but it will still be wrong and unacceptable that people should have to live under a regime that lacks the legitimacy that characterises a MV.

If, on the other hand, the legitimacy that figures in MV is more empirical in character, its normative significance will be less direct. It will contribute to the general notion that to be a MV an arrangement has to be one that the interested parties accept, albeit often with reservations and some reluctance, and the greater the degree of empirical legitimacy that a MV enjoys, the better it will function as a MV. But legitimacy will be instrumentally rather than intrinsically significant; it will have value because it facilitates the goods that MV makes possible, especially the goods of peace and security.

In some measure, Horton himself leaves the issue of whether the political theory of MV should be normative in purpose as an open question and one that is subsumed by the larger issue of what should be the character and aim of the realist political theory that he and others are seeking to develop (2010a, 444–6). However, the burden of his published remarks is that his political theory of MV is not straightforwardly normative in purpose. He accepts that the idea of MV will contain an element of normativity simply because so many of the concepts central to politics, such as ‘coercion’ and ‘legitimacy’, are permeated with normativity. But to acknowledge that the political theory of MV will inevitably be, like any other political theory, ‘normatively inflected’ is not to accept that it should be directly prescriptive in purpose. While he does not rule out altogether the possibility that realist political theory might be prescriptive, he is more inclined to doubt that such prescription is consistent with the realist’s scepticism about the capacity of political theory to guide the conduct of politics. His political theory of MV, he says, ‘does not aim to provide much by way of practical guidance’ (2010a, 445). It would seem to be more interpretative than normative in purpose. In that respect his theory of MV differs from that of some others, who share in the prescriptive purpose of mainstream liberal political theory even though they are more modest than the likes of Rawls in what they recommend (e.g. Neal 1997; McCabe 2010). Horton’s account of MV aims not merely to invest the recommendations of liberal moralism with a more realistic content; it is part of a larger endeavour to reshape the character and purpose of political theory.

## Why Modus Vivendi?

Before turning to assess Horton’s thinking on MV, there is a further question we might ask about it. Why does he think that MV should figure so pre-eminently in our thinking about the nature and potentialities of political life? The answer is not self-evident, nor need it be the same for all proponents of MV. Apart from Horton, the political theorist who has done most to develop the idea of MV is John Gray (2000). Gray’s advocacy of MV is the offspring of his commitment to value pluralism. ‘Value pluralism’ here does not describe the mere fact that different people possess different and often conflicting ideas of what is valuable. Rather it is a meta-ethical thesis about the nature of value itself: the thesis that values are themselves conflicting, sometimes uncombinable and frequently incommensurable, so that we can identify no one value (such as human utility) as the supreme value, nor any particular configuration of values as the uniquely right configuration. When that thesis is applied to forms of life, it entails that there are many different good forms of life, none of which can be identified as better than the rest, and not all of which can be realised in a single life or in a single community. For Gray, MV is the best we can do in politics, because political institutions have to cope with the diversity of commitments and forms of life that are the inevitable consequence of value pluralism, but also because value pluralism applies to political institutions themselves – there is no one political system that we can identify as uniquely just or uniquely legitimate.

While Horton has some sympathy with value pluralism as a thesis about the conflictual nature of values, he dissents from Gray’s efforts to make it the foundation for MV (2007, 43–49; 2011a 123). Value pluralism is a controversial thesis and its claims about the nature of value are at odds with those of many other prominent types of moral thinking, such as religiously-based moral beliefs. Rather than providing a plausible and realistic foundation for MV, it belongs in the mix of different and conflicting views for which MV has to provide. Horton also faults Gray for taking too little account of disagreement over the basic distinction between good and bad and for neglecting sources of conflict other than value pluralism, such as differences of interest.

Gray does exhibit some of the scepticism that lies at the root of Horton’s commitment to MV but only insofar as value pluralism exposes certain aspirations and ways of thinking as wrong-headed. Horton’s scepticism is closer to the ground and more thorough-going. He locates the political theory of MV within the sceptical and anti-utopian school of thought that he and others describe as ‘realism’. Why? One answer would seem to be modesty about what we can expect from human beings. We may glimpse a better world but we err if we suppose that human beings are capable of sustaining it. Real people will never match Rawls’s idealised citizens; they are not capable of the highly moralised and consistently just conduct that Rawls requires of them. For Horton, familiar truths about ‘human weakness and fallibility’ make the case for the politics of MV more powerfully than does Gray’s value pluralism (2007, 55; 2010a, 434).

However, that is not where the only or perhaps the main burden of his case lies. Another factor that is important in his thinking is contingency (Horton 2005, 31; 2010a 436, 439–40, 2011a, 124). In politics we never face clear, clean circumstances and we never start from a blank slate. We always inherit a particular context and particular circumstances of which we can only make the best. The sheer contingency, untidiness and incongruity of those circumstances preclude the realisation of neat and symmetrical models of the just society.

But that is not the whole story either. As we have already noticed, Horton reserves a special scepticism, sometimes little short of contempt, for ‘moralism’ and particularly ‘liberal moralism’, which is the pejorative label he uses to describe the deontological liberalism of Rawls, Charles Larmore, Ronald Dworkin and Brian Barry and, we might add, Gerry Cohen, Amartya Sen, Thomas Nagel, Thomas Scanlon and Thomas Pogge; in fact, a very large chunk of the major names that have emerged in Anglo-American political philosophy during the last half century.

What is wrong with ‘moralism’? Part of the answer lies in elements of Horton’s thought that we have already touched on: over-idealised expectations of human beings and over-idealised and insufficiently particularised conceptions of the circumstances that we confront in politics. But there are other answers too. One is that moral theory, or a moral theory, simply will not provide all of the answers to all of the issues and circumstances we confront, especially in politics. Horton appears to think that we exhaust the resources of morality sooner than the moralists think and we are quickly reduced to dealing with issues in non-moral or amoral terms, or at least in terms on which moral theories are indeterminate or ambiguous. Another answer is that morality is itself so frequently a matter of dispute that, rather than providing the solution, it forms part of the problem. The Rawlsian solution to disagreement is to resort to principles of right that stand separately from conflicting conceptions of the good and the comprehensive doctrines from which those conceptions derive, so that we can use a higher level political morality to manage the conflicts that occur lower down the moral hierarchy. Horton’s response is to observe that there is no higher level morality that is immune from conflict. Disagreement can go all the way up as well as all the way down; there are no common or higher-level principles to which people can reliably turn to resolve their disagreements (2003, 2010b). They have simply to make the best of the conflictual circumstances in which they find themselves and to do so with whatever resources they can find within those circumstances rather than by looking outside or above them.

Against that background, it is unsurprising that, whilst Horton makes legitimacy a feature of MV, it is a legitimacy that is highly particular to the context and culture of each society and highly dependent on what, as a matter of fact, the relevant population finds acceptable. It is equally unsurprising that morality figures in such a muted and secondary way in his account of MV. His thinking on MV may not dispense with morality altogether (2007, 52–3; 2010a, 441), but it is certainly intended as an antidote to moralism.

## MV and Actual Political Systems

Horton’s thesis on MV is so broad-ranging and raises such large and fundamental issues about the nature of politics and political theory that my comments on it in the remainder of this article will be no more than limited, piecemeal and inconclusive. The general burden of what I have to say questions whether Horton is overly sceptical about what is possible both in politics and in political theory.

One way of testing Horton’s claims about MV might be to examine how far current political systems conform to his model of MV. MV is especially associated with conflict and the resolution of conflict and there are plenty of instances in the modern world of regimes that bear the marks of the divisions and disputes they have had to manage. We might think, for example, of the consociational arrangements that characterise the Belgian and Swiss political systems, or the form that federalism has assumed in Canada in response to division between the French and English speaking populations, or the way in which representation in the Fijian Parliament has been structured to reflect the country’s different ethnic communities. The power-sharing arrangement that has existed in Northern Ireland since 1998 is a classic instance of a MV that has secured a fragile and hard-won peace in a context of bitter and previously bloody conflict. Horton himself cites the Good Friday Agreement as a paradigm instance of MV (2011a, 132–4).

However, my understanding of Horton’s political theory of MV is that it is not intended to provide only for the special circumstances of ‘deeply divided’ societies, such as Northern Ireland. The model of MV is meant also to capture the character of political systems that exist in more homogeneous societies and whose politics assume a more ordinary workaday character. If the reality is that we can never do better than a MV, it must be the case that no actually existing regime has managed to be more than a MV, although it may fall short of a MV as in the case of tyrannies and failed states. Is that the reality of our world? This is where lack of precision in the idea of MV becomes a problem. MV requires there to be some consensus, but not too much, otherwise we shall go beyond a MV. So how do we test the empirical claim implicit in Horton’s political theory of MV? One possibility is to go back to the ideas of disagreement, dissatisfaction and compromise. A population is involved in a MV if its members accept a political arrangement and bestow upon it some measure of legitimacy, but still regard as less than wholly satisfactory and as falling short of what there really ought to be. If they come to regard the arrangement as *wholly* right, if they are *fully* or *very* content with it, if they see no reason to change it even if they could, then they would seem to have arrived at an arrangement that is more than a MV.

If that is the test, and if we look around at reasonably stable European societies like Britain and Denmark and nowadays Germany, and at the US – the kind of societies Rawls means to address in his theory of justice – it is not clear that they are accurately described as MV. That is not to say that they match Rawls’s model of a just society but only that, as a matter of fact, consensus on the basic structure of the political system seems to be broader and deeper than is exhibited by the parties to Horton’s MV. It is perhaps rare for every aspect of a society’s basic structure to be entirely free from dispute. Think, for example, of disagreements over the electoral system and the possibility of Scottish independence in Britain. But that sort of particularised dissensus can exist alongside much that is settled and undisputed.

Another factor that is relevant to the match between the political world and Horton’s account of MV is the *level* of a society’s politics at which MV is supposed to exist. Most discussions of MV, including Horton’s, contemplate MV at the very foundation of a society and that is the sort of MV to which my comments have been addressed. But Horton also discusses MV in relation to the Rushdie Affair and the issue of abortion (2003, 17–21; 2007, 53) and those issues move us more into the realm of ordinary politics. Certainly the more one ascends from constitutional fundamentals to ordinary everyday political issues, the harder it becomes to deny the reality of disagreement, dispute and contestation. If we describe those ordinary political differences and their resolution through established political processes as manifestations of MV, MV will be an omnipresent feature of politics. But if we make MV synonymous with politics itself, we deprive it of any significant independent meaning. That is not to deny that there may be particular political issues that divide a society so deeply that they are not easily contained by the society’s ordinary political processes, and any would-be resolution of them will rank, for the divided parties, as no more than a MV. But that is very different from the ordinary run of political disputes and disagreements that all sides accept are rightly resolved through their political system’s decision-making processes. If, however, we reposition MV at a more fundamental level so that its concerns are constitutional, I am not persuaded that what we find in our world is, always and everywhere, arrangements that are, for those whom they govern, never more than a MV.

Yet that verdict may result from subjecting MV to the wrong sort of test. Although conflict amongst a population figures prominently in Horton’s account of MV, that conflict may need to be neither persistent nor current. A society may have worked out institutions for dealing with its divisions long ago and its current generation of citizens may be heir to none of the discontent or sense of compromise that has accompanied the historical development of those institutions. They may accept their inherited political system as simply ‘their’ way of doing things, which they embrace with little or no dissatisfaction and perhaps with little or no reflection. Nevertheless, Horton is still likely to regard their political system as a MV, which suggests that its character as a MV turns not on how deeply and thoroughly the relevant population accepts it, but on the nature of the arrangement itself. It is the product of all sorts of historical circumstances, contingencies and compromises and it remains a creature of its origins. That, rather than instantiations of political theory or abstract principle, is the reality of political systems. Just as Michael Oakeshott held that in politics, whatever we may think we do, we never actually do any other than pursue intimations, so Horton’s claim may be that, no matter how we may think of political arrangements, they never actually amount to more than MV. And just as Oakeshott understood ideological politics to rest upon a misconception of the nature of politics and of itself, so Horton seems to believe the general run of contemporary political philosophy, especially liberal moralism, to rest upon a misconception of the nature of the political reality for which it seeks to provide.

## Modus Vivendi and Contemporary Political Theory

Is Horton right to be quite so dismissive of the type of political philosophy that he brands ‘liberal moralism’? Much of his critical fire is directed at the work of Rawls, understandably since Rawls is generally recognised as the principal standard bearer for what Horton calls liberal moralism. I begin therefore by commenting on Rawls’s political philosophy in relation to Horton’s critique. The issue I want to take up, however, is not whether the substance of Rawls’s argument is correct but whether the kind of exercise he is engaged in is so remote from political reality as to be wrong-headed and pointless.

It is easy to exaggerate the extent to which Rawls’s political thinking is idealistic. In many ways, Rawls, especially the ‘later’ Rawls, is a very conventional thinker. Recall that his political liberalism and his political conception of justice are based on fundamental ideas that he takes to be already present in the public culture of democratic societies – ideas of society as a fair scheme of cooperation, of persons as free and equal, and of well-orderedness (Rawls 1993). He has primarily in mind the public culture of the US, but I understand him also to include the public cultures of other societies that are normally described as liberal democratic. He ‘works up’ his principles of justice from fundamental ideas that are already present in those public cultures. Horton, unsurprisingly, contrasts Rawls’s thinking unfavourably with Oakeshott’s politics of scepticism (2005, 31–2), but Rawls’s own approach has an Oakeshottian character – he understands himself to be pursuing what is already intimated in the public cultures of the societies for which his political conception of justice is designed. Rawls’s account of a just international order (1999) is even more strikingly conventional and limited in ambition than is his theory of justice for a liberal democratic polity. He describes the just order that he sketches for the international world as a ‘realistic utopia’, but it has struck many commentators, particularly those with more cosmopolitan inclinations, as decidedly more realistic than utopian (cf. Martin and Reidy 2006).

One thing that encourages the perception of Rawls’s thinking as idealistic is the distinction he makes between ideal and non-ideal theory and his representation of his own work as primarily an exercise in ideal theory. The very notion that there is scope for a distinction between the ideal and the non-ideal in politics is often scorned by realists, who believe that it betrays either naivety or a wilful and pointless failure to confront the realities of the political world. Horton, himself, observes that the distinction is one ‘of which the political theory of modus vivendi is naturally suspicious, and for which it has little use’ (2011a, 126). However, the purpose of the distinction is easily misunderstood and actually has very little to do with ‘utopianism’ as that term is ordinarily understood. It is designed to distinguish between two sorts of question:

(i) What should be the basic structure of our society, whether it be a domestic society or the international society of peoples? What should be the basic rules and arrangements of that society? That is the concern of ideal theory.
(ii) What should we do when things go wrong – when people (either ordinary citizens or their governments) do not comply with those rules and arrangements? That is the concern of non-ideal theory. For Rawls, non-ideal theory is primarily about non-compliance; it is about issues such as crime, punishment and civil disobedience in the domestic case, and outlaw regimes, ‘burdened societies’, and just war in the international case.

The distinction between ideal and non-ideal theory is not therefore a distinction between more idealistic and more realistic political theory. Rather, it badges two different sorts of issue and signals a division of labour in our moral and political thinking.

The ‘ideal’ of ideal theory need not therefore be highly ‘idealistic’ and utopian in character. On the contrary, the ideal of ideal theory can take full account of the limits that constrain what we might reasonably expect to achieve. It is perfectly possible, for example, to apply the ideal/non-ideal distinction to a thinker like Hobbes, whose name is often associated with MV (e.g. Neal 1997, 185–201). His *Leviathan* is primarily an exercise in ideal theory; it sets out the type of regime that human beings would ideally construct. It also describes the type of regime with which they would always comply if they were fully and constantly enlightened about their self-interest. Unfortunately, some will be foolish or ignorant, or they will be led astray by false doctrines, and so the sovereign, as well as playing the role ascribed to him in ideal theory, has to cope with the non-ideal circumstance of non-compliance. But the issue of what sort of authority there should be and what sort of role it should perform is for Hobbes, in the first instance, an issue of ideal theory. The fact that we would never describe Hobbes’s political prescriptions as utopian does not make them non-ideal in Rawls’s sense.

For Rawls, the idea of a MV forms part of his story about the development of an overlapping consensus on the principles of a just society. That story is most readily understood as one about how the political toleration of religious differences in western societies – primarily differences within Christianity – evolved in the centuries following the Reformation. It begins with a MV, a mere stand-off grounded in people’s weariness with war and bloodshed but, over time, the settlement develops into something more. People come to think of religious freedom as a right and they increasingly abandon the idea that political power should be used as an instrument of religion. So we have a broad consensus developing that public provision for people’s religious differences should be governed by principles of freedom and equality. Although presented in a simplified form, that story is quite plausible. Most people in western societies who have a religious faith do not now view religious freedom and equality as poor substitutes for the confessional state; it is not for them a reluctant compromise. It is how things really ought to be in a religiously diverse society. Not everything has been settled; issues such as abortion and marriage and limits on free expression remain matters of dispute amongst those of faith and of no faith. But the commitment to religious freedom, which Rawls treats as a ‘settled conviction’ (1993, 8), and the wrongness of using political power to impose or to promote a particular faith or denomination, are now matters of widespread consensus.

It is fair to add that the breadth and depth of consensus Rawls hopes for from his model citizens set the stakes high and arguably unnecessarily high. First, his overlapping consensus is a consensus of ‘comprehensive doctrines’, which include moral and philosophical as well as religious doctrines, and the consensus that has developed around the proper relationship between religion and political power does not yet have a clear equivalent for non-religious doctrines. Secondly, Rawls often speaks as though he expects his citizens not only to embrace his conception of justice as a free-standing political conception, but also to find positive reason for endorsing it within their different comprehensive doctrines. That is probably both implausible and unnecessary. I suspect, for instance, that the great majority of people living in western liberal democracies who have religious beliefs make little if any connection between those beliefs and the basic political structure of the societies in which they live, although some do. Thirdly, the stability that preoccupies Rawls is *intellectual* stability as much as *political* stability. For example, amongst the comprehensive doctrines that concern him are utilitarianism and Kantianism and he frets over whether the adherents of those doctrines will be able, without inconsistency, to embrace his political conception of justice. To date there has been little reason to translate that philosophical worry into a political worry; the destabilising social conflicts with which politicians have had to cope have not, so far, included those coming from the massed ranks of discontented utilitarians and Kantians. On the other hand, these critical observations on Rawls’s ambitions for an overlapping consensus are not necessarily grist to Horton’s mill. They suggest that getting people who possess different comprehensive doctrines to accept a common set of political principles may be less difficult in practice than Rawls makes it in theory.

There are, however, other sorts of division, other forms of pluralism, that create real problems of stability which may be major concerns for the theorist of MV but in which Rawls shows little interest. These are divisions and conflicts that arise out of differences of nationality, ethnicity, language, and also religion in cases where it is different religious *identities* rather than different religious *doctrines* that foment social and political conflict. In dealing with those differences, MV may be very much to the point. Here I want to comment briefly on how the differences between doctrinal conflict and identity conflict relate to Horton’s critique of Rawls.

Rawls is concerned with comprehensive doctrines because those doctrines have normative content. If they were truly comprehensive they would have a normative content that covered every aspect of life, including politics. In fact, none is fully comprehensive and so we find Rawls using the oxymoronic phrase ‘partial comprehensive doctrine’. But even a partial comprehensive doctrine may contain norms that prescribe or imply a political order different from Rawls’s political conception of justice. That is why he has to worry about whether his political conception of justice can secure a consensus amongst people who embrace different comprehensive doctrines. But, where differences are differences of *identity*, they are not necessarily accompanied by the sort of *doctrinal* differences that create the potential for conflict over what is a just political system. Identity differences may be no less problematic for political stability than doctrinal differences, and they may often be more problematic, but they are problematic in a different way.

Secondly, insofar as identity differences generate conflict, that conflict may have a different political focus. Consider the case of nationality and Northern Ireland. In the history of Ireland, conflicts of religious belief have been extremely important but the recent Troubles have not been primarily conflicts over religious doctrine; IRA and Loyalist paramilitaries have not killed members of one another’s community, and often members of their own, because they disagreed over matters of theology. Simplifying drastically, the recent conflict has been between different national identities which are closely associated with different religious identities. Loyalists and unionists have wanted Ulster to remain part of the UK; republicans and nationalists have wanted Ulster to form part of a united Ireland. What the conflict has *not* been about is the sort of political system under which they should live. It was fundamentally about what their political *unit* should be, rather than about Rawls’s concern – what their favoured unit’s *political system* should be. There is no reason to believe that unionists and nationalists wish to live under fundamentally different sorts of political system, any more than do the citizens of Great Britain and the citizens of the Republic of Ireland. Thus, the disagreements and conflicts that concern the political theorist of MV, or to which a particular MV is addressed, need not be those that preoccupy Rawls.

Given the realities of the contemporary world, the political theorist of MV might well criticise Rawls for focusing on the wrong sort of pluralism, and some do. Claudia Mills, for example, argues that Rawls’s neglect of issues of race, gender and ethnicity helps him to overlook the case for MV (Mills 2000, 203) and Jacob Levy suggests that, when we add ethnic, cultural and linguistic pluralism to religious pluralism, the case for MV is more compelling than Rawls recognises (cf. Levy 2007, 190–3). It is certainly true that the principles Rawls develops for dealing justly with a plurality of comprehensive doctrines are often not easily re-applied to other forms of pluralism. On the other hand, that is to point out the limits of Rawls’s endeavour rather than to establish its irrelevance or impracticability, and issues of racial, ethnic, cultural and linguistic difference, and differences of national identity, have hardly been neglected by other liberal political philosophers.

Horton does, however, charge liberal moralism with ignoring other major questions and he links that neglect to its moralistic preoccupations and its failure to allow its agenda to be shaped by the real world of politics. The many real-life political subjects on which, he complains, liberal moralism has had little if anything to say include political judgement, leadership, representation, political responsibility, what is politically possible, and how the transition from currently existing societies to the much vaunted just society might be effected (2009; 2010a 437, 445). For some of these subjects, e.g. political representation, bibliographies might be compiled in defence of liberal political theory, but for others the charge is hard to deny. Liberal political theory’s enthusiasms are undoubtedly accompanied by blind spots. It may be, however, that liberal political philosophy is sometimes relatively silent on particular subjects as a consequence of the very modesty of ambition that Horton urges upon it. Take the case that Horton has complained about at greatest length: political leadership (2009). It may be that liberal and other normative forms of political theory have said little about leadership, not because their practitioners have failed to recognise its significance in politics (could they really have been so purblind?) but because, as normative political theorists, they have recognised that their competence to say much of consequence on that subject is very limited. If they were to attempt to instruct presidents, prime ministers and other politicians in the art of leadership or in the exercise of political judgement, Horton would scorn their presumption and naivety – and rightly so. Normative political theory’s uneven concern with political issues may reflect not its own unworldliness and self-obsession but rather its recognition that, while there are some aspects of politics to which it can make significant and valuable contributions, there are others on which it has little competence.

Of course, that takes us onto the question of what the task of political theory should be. As we have seen, Horton gives little normative or prescriptive *purpose* to the political theory of MV, which presumably means that he has little, if any, normative or prescriptive aspirations for political theory in general. But if political theory ceases to be normative in concern, even though it might continue to be ‘normatively inflected’, it is difficult to see what substantial purpose it might have. It can, of course, continue to engage in conceptual analysis, but Horton’s recasting of political theory in a realist mode seems to project more for it than that. It is just not clear what that ‘more’ might be. Take the case of leadership again. In attacking liberal moralism for neglecting leadership, Horton implies that leadership is a subject on which political theorists can and should have something significant to say. But what might that be? It is difficult to think of an answer that would not entail political theorists usurping the domains of other sorts of scholar, such as empirical political scientists or political sociologists or political psychologists, and surely the last thing we want is a return to the ‘armchair political science’ of many decades ago, when people purported to tell us all about the political world while never leaving their studies to investigate it. There are issues relating to leadership that are obvious topics for political theory such as the issue of ‘dirty hands’, but that issue is both ‘much discussed’ and normative through and through, and it does not cease to be normative just because we recognise the inadequacy of jejune forms of moralism.

## Conclusion

My purpose in this article has not been to debunk the case that Horton makes for MV. Quite the contrary, his articulation of the nature of MV and of the need to recognise MV as a central feature of political life constitutes one of his most significant contributions to political theory. Although he is not alone in defending MV, he has done more than anyone else to investigate and develop the substance of that idea. He also gives a compelling case for understanding the political world through the idea of MV and makes sense of the reality that chaos and repression are not the only alternatives to a just, or nearly just, society. His emphasis on contingency, compromise and circumstance is a much needed reminder that we have to take the world as we find it and make the best of what we find. Unlike Plato’s philosopher-rulers, we cannot begin by scraping the canvas clean and, even if we could, we could not complete the picture by using the resources of political theory alone.

The major doubt I harbour is whether Horton’s understanding of the political world in terms of MV is really as corrosive of contemporary political philosophy, including ‘liberal moralism’, as he appears to believe. Do we have to be so downbeat about the politics of MV that the standard concerns of political philosophy evaporate into irrelevance? It is hard to accept that the issues a political philosopher like Rawls takes up – the freedoms that should characterise a liberal society, the just distribution of income and wealth, the proper scope of political power, etc. – are not real political issues or that carefully considered analysis and argument on those issues is of little practical relevance to how we should organise our political lives. Even a MV can be better or worse, more or less just. Indeed, if we cleanse the term ‘modus vivendi’ of the pejorative associations Rawls attaches to it, it requires no great stretch of usage to describe his own political conception of justice as an exercise in MV – an effort to work out a ‘way of living together’ that the members of a plural society have reason to accept is fair. The ‘idealised’ method he uses is not idealised in a way that deprives its outcomes of any evident application to the societies in which we live.

However, Horton’s scepticism about normative political theory extends far beyond Rawls and his theory of justice. The nature of his critique of liberal moralism suggests that his scepticism takes in all other forms of political theory that are significantly normative in purpose. I remain baffled by the notion that politics is so *sui generis* that (what is sometimes called) ‘practical philosophy’ loses its capacity to be practical when it turns to politics. Horton is, of course, right to observe that political theory has a tendency to become insular and to feed off itself (2009, 18), but that sort of disciplinary introversion is perhaps an inescapable hazard of academic life. If we turn to economics or sociology or ‘IR’, we find disciplines that are no less introverted and self-absorbed. But the basic normative questions that drive contemporary political philosophy are real questions and it is hard to see how, even in the political world of MV, they can become unreal. If political philosophers cease to examine them, they will not go away; and they will not go away precisely because they are pressing practical questions. Others will still try to answer them and will probably do so even less adequately.
